# Are Routine Postoperative Hemoglobin Tests Justified in All Patients Who Undergo Total Hip Arthroplasty Due to a Displaced Femoral Neck Fracture?

**DOI:** 10.3390/jcm13154371

**Published:** 2024-07-26

**Authors:** Shanny Gur, David Segal, Alex Tavdi, Yuval Fuchs, Dan Perl, Alon Fainzack, Nissim Ohana, Michael Markushevich, Yaron Shraga Brin

**Affiliations:** Department of Orthopedic Surgery, Meir Medical Center, Tel-Aviv University, Kfar-Saba 4428164, Israel

**Keywords:** displaced femoral neck fracture, total hip arthroplasty (THA), tranexamic acid (TXA), blood loss, fast tracking

## Abstract

**Background:** Total hip arthroplasty (THA) is a standard treatment for a displaced femoral neck fracture in the elderly. In contemporary healthcare, there is a global shift towards fast-track treatment modalities, prioritizing early hospital discharge for patients. Consequently, routine postoperative blood tests may become redundant, offering significant time and cost savings. We aim to evaluate postoperative hemoglobin levels in trauma-related THA cases and identify patient profiles for whom these tests hold significance. **Methods:** A retrospective review of 176 THA procedures performed between 2018 and 2022, focusing on individuals undergoing THA for displaced femoral neck fractures. Multivariable logistic regression analysis was employed to identify factors associated with postoperative hemoglobin levels below 8.5 g/dL. **Results:** Of the 176 patients included, 109 (61.9%) were women and the mean age was 69.09 ± 8.13 (range 27 to 90) years. The majority of the patients underwent surgery within 48 hours of admission. The mean preoperative hemoglobin (Hb) level was 13.1 ± 1.4 g/dL, while the mean postoperative Hb level was 10.5 ± 1.2 g/dL. Only six patients (3.41%) exhibited postoperative Hb levels of ≤8.5 g/dL. No significant associations were found between postoperative Hb levels ≤ 8.5 and any demographic, surgical, or medical characteristics. **Conclusions:** Our findings suggest that routine postoperative blood count testing may not be necessary for most patients undergoing THA for displaced femoral neck fractures, particularly those without complications or significant comorbidities.

## 1. Background

Hip fractures pose a significant global public health challenge, with around 1.5 million cases reported annually worldwide. This number is expected to soar due to aging populations, with projections foreseeing a rise to 2.6 million by 2025 and a staggering 4.5 million by the 2050s [1]. Nearly half (45–50%) of all hip fractures occur in the subcapital (femoral neck) region, with the majority being displaced. Total hip arthroplasty (THA) is a frequently utilized surgical intervention in modern orthopedic practice, offering an effective treatment solution for these fractures [2,3,4].

Following major orthopedic surgeries, including THA, blood tests are standard practice as part of postoperative protocols and aid in clinical decision-making [5]. These assessments are essential for managing acute and chronic conditions and are routinely ordered postoperatively to identify potential severe complications. THA is traditionally associated with substantial blood loss, necessitating blood transfusions in 11 to 21% of patients [6,7].

In recent years, there has been a notable trend toward implementing fast-track surgical pathways in orthopedic surgery, aiming to minimize perioperative complications and reduce hospitalization costs. A key strategy within this approach involves the administration of tranexamic acid (TXA) to reduce bleeding, expedite patient discharge, and accelerate postoperative recovery, particularly in cases where a fast-tracking approach is implemented [5]. The fast-tracking approach aims to optimize each step of the perioperative journey, thereby reducing hospital stays, associated costs, morbidity, and the risk of complications while improving patient experience. These multifaceted improvements contribute to cost reduction within the healthcare system [8,9,10].

However, delays in hospital discharges frequently occur while awaiting routine blood test results following total hip arthroplasty (THA). This has prompted discussions regarding the necessity of routine blood tests shortly following THA [11,12].

The aim of this study was to evaluate the necessity of routine postoperative laboratory testing in patients undergoing unilateral THA following a displaced femoral neck fracture shortly following the operation while also exploring the feasibility of implementing fast-tracking protocols. 

## 2. Methods

This retrospective cohort study was conducted using the STROBE checklist [13]. The study protocol was approved by the hospital’s Institutional Review Board—MMC 0327-20. Date of approval—10 November 2022.

### 2.1. Patients

Our electronic health records (EHR) from 2018 to 2022 included data on 458 patients who underwent THA at our institution. The inclusion criteria were active individuals who underwent THA in the direct anterior approach [14] due to a displaced femoral neck fracture. Notably, the majority (80.7%) of patients underwent surgery within the first two days of admission, with 13.1% undergoing the procedure on the third day and the remaining later on. Following the application of the inclusion criteria, our final cohort comprised 176 patients, including 109 (61.9%) women and 67 (38.1%) men, ranging in age from 27 to 90 years. Patient selection for THA adhered to the criteria outlined in the NICE guidelines, encompassing ASA grade 1–3, ability to walk independently with no more than a cane, and mental acuity [15].

### 2.2. Surgical Approach

#### 2.2.1. Direct Anterior Approach

All patients within our cohort underwent surgery using the direct anterior (DA) approach to the hip joint. They were positioned supine on a standard operating table with adjustable hip flexion. We used the modified Hueter approach to the hip joint, beginning with a transverse skin incision on the anterior aspect of the thigh, starting 5 cm distal to the anterior superior iliac spine and extending up to 10 cm laterally on the anterior aspect of the thigh. The DA approach entailed exposing the tensor fascia lata with subsequent division of its perimysium. The lateral head, or reflected portion of the rectus, was retracted medially utilizing the interval between the sartorius and tensor fascia lata. Subsequently, we performed an anterior capsulotomy to expose the fractured femoral neck. An osteotomy of the femoral neck followed, with extraction of the femoral head and neck using a corkscrew. Routine preparation of the acetabulum and femoral canal ensued, with particular attention to preserving the abductor mechanism and short external rotators through selective soft tissue releases on the posterior aspect of the femoral neck.

#### 2.2.2. Implants

All patients underwent THA using the Corail cementless stem and Pinnacle cementless cup (Depuy Synthes, Warsaw, IN, USA).

#### 2.2.3. Surgical Team

All patients underwent surgery performed by the same surgical team. The team included the same three senior surgeons from our arthroplasty unit, each with extensive experience performing hundreds of cases using the direct anterior approach to the hip joint.

#### 2.2.4. Measurements

Data abstracted from the electronic health records (EHR) included an array of variables, including demographic information (age, gender), comorbidities, indication for THA (osteoarthritis (OA) or displaced femoral neck fracture), surgical data (duration, surgeon’s expertise level, date, procedure code, type of anesthesia), preoperative and postoperative hemoglobin (Hb) levels, postoperative blood transfusions, and hospitalization details (length of stay, date of discharge, etc.).

#### 2.2.5. Data Analysis

We utilized descriptive statistics to present patient demographics and medical features. The target parameter was preoperative hemoglobin level, and the secondary parameters were the patient, pathology, and treatment features as possible predictors of the outcome measure, a postoperative hemoglobin level < 8.5 g/dL. For the univariable analysis, we studied categorical variables using a Fisher exact test. Each continuous variable was tested for normality with a Shapiro–Wilk test. For variables with a normal distribution, a Student’s *t*-test was utilized, whereas continuous variables with a non-normal distribution were studied using a Mann–Whitney U test. When deemed proper, continuous variables were divided categorically to increase reliability and preciseness. The duration from admission to surgery was presented in this method. We then conducted a multivariable logistic regression analysis to determine factors associated with hemoglobin levels < 8.5 g/dL. *p*-value of 0.05 was set for statistical significance. A receiver operator curve analysis was used to identify the optimal cutoff when a continuous variable was found to be associated with a categorical outcome. SPSS 28.0 software (Armonk, NY, USA, IBM Corp.) was used to analyze the data.

## 3. Results

A total of 176 patients were included, 109 (61.9%) women, with a mean age of 69.1 ± 8.1 years (range, 27 to 90). Preoperatively, the mean Hb level was 13.1 ± 1.4 g/dL (range, 9.6 to 17), while postoperatively, the mean Hb level was 10.5 ± 1.2 g/dL (range, 7.4 to 14.2). The mean decrease in Hb levels postoperatively was 2.5 ± 1.3 (range, −1.1 to 7.4) g/dL. Among the study participants, 170 patients (96.59%) maintained postoperative Hb levels above 8.5 g/dL (Table 1).

Univariable analysis by postoperative Hb levels of 8.5 g/dL or higher is presented in Table 1. None of the assessed variables were found to be statistically significant.

A total of 33 (18.75%) patients received at least one packed red blood cell transfusion perioperatively, with 30 (17%) patients receiving at least one transfusion postoperatively and 3 (1.7%) patients during surgery or pre-operatively. Patients who received postoperative transfusions exhibited lower postoperative Hb levels when compared to those who did not (9.4 ± 1.1, range, 7.4 to 12.5, vs. 10.8 ± 1, range, 7.5 to 14.2 g/dL, *p* < 0.001). Five (83%) patients in the Hb ≤ 8.5 g/dL group received a transfusion, compared to 28 patients (16.4%) in the second group (Table 1). Nevertheless, a transfusion was not associated with patient demographics or medical characteristics (*p* > 0.05). The mean preoperative Hb was lower in patients who were transfused postoperatively (Table 2). A ROC analysis that plotted preoperative hemoglobin against blood transfusion had an area under the curve (AUC) of 0.61 with a lower 95% confidence interval (overall model quality) of 0.51, and a maximal Yoden index of 0.21. These measures reflected a poor model quality that provided only limited ability to predict the outcome variable. In this limited model, a preoperative hemoglobin level cutoff of 12.25 g/dL provided the highest Yoden index (0.21) with a sensitivity of 45.5% and a specificity of 75.5% for predicting the administration of a blood transfusion. Table 2 summarizes the characteristics of patients who received blood transfusions.

## 4. Discussion

In the context of advancing rapid recovery in THA surgery, there is a need to reassess the necessity of routine postoperative blood tests. While potentially informative, these tests may inadvertently contribute to discharge delays, prompting a reevaluation of their utility. Recent studies have evaluated blood loss in elective total knee and total knee surgeries [16,17,18,19,20,21]. This study aimed to discern the circumstances under which postoperative blood tests can be omitted, following routine THA for femoral neck fractures, and when their necessity remains warranted. Our findings indicate that only a minor percentage of this patient population required postoperative blood tests and hemoglobin monitoring, as most patients exhibited normal postoperative hemoglobin levels. Thus, even in non-elective procedures such as THA for displaced femoral neck fractures, the majority of the patients can follow the fast-tracking protocols and be discharged on the day of surgery without waiting for blood test results.

Fast-track THA represents an approach geared towards optimizing patient outcomes and accelerating recovery post-surgery. This protocol offers numerous advantages. By reducing hospital stays, it promotes cost-saving benefits both for the patients and the healthcare providers [22]. Employing fast-track pain control protocols aims to alleviate postoperative pain by using multimodal pain management strategies, potentially facilitating faster recovery with reduced pain levels [8]. Evidence indicates that fast-track THA contributes to shortened recovery periods, enabling patients to return to their preoperative activities faster, often with high satisfaction levels [23]. However, it is essential to acknowledge the potential risks and disadvantages associated with fast-track THA. Persistent pain, swelling, and difficulty in rehabilitation have been reported by 5–15% of patients [24]. Some patients have been noted to under-consume analgesics, which may contribute to pain and swelling [25]. Overall, there is compelling evidence that an accelerated perioperative care and rehabilitation protocol can be both cost-saving and clinically more effective after total hip arthroplasty [22].

We conducted a comprehensive review of existing studies that explored the need for postoperative laboratory tests and other factors associated with blood loss following THA, such as BMI, surgical techniques, comorbidities, gender, age, and more.

Yang et al. [5] established that female gender was a risk factor for blood transfusion. However, our study yielded contrasting results as we found no significant correlation between gender and hemoglobin loss or the need for blood transfusion following THA in the studied population.

Carling and coworkers demonstrated that low BMI poses a risk factor for excessive blood loss in hip and knee arthroplasties [26]. Conversely, Prasad et al. and Hrnack et al. [16,17] found no correlation between higher BMI and blood loss. Consistent with their findings, our investigation also found no significant relationship between BMI and blood loss.

Walsh et al. reported that elderly individuals tend to bleed more following THA [19]. These findings could be attributed to medication use, underlying health conditions, etc. Miao et al. found a negative correlation between age and blood loss in THA [18]. In our study, however, we failed to observe any significant relationship between age and blood loss, as patients across both groups were of similar ages.

It is well established in the literature that the duration of surgery is positively correlated with bleeding [20,21]. Interestingly, the duration of surgery was similar in both groups, and we could not establish a correlation between blood loss and the length of the surgical procedure.

In cases where a patient’s Hb level drops below 8 g/dL, blood transfusions are commonly recommended [7,27,28,29]. It is well known that Hb levels tend to decline until the fifth postoperative day [30]. In our study, we focused on treating fragile elderly patients with multiple comorbidities, while the femoral neck fracture serves as the “tip of the iceberg” in those patients. These are not the healthy elective patients who undergo THA for hip osteoarthritis. For those reasons we set the cutoff for analysis at an Hb level of 8.5 g/dL. Similarly to our findings, other surgeons have opted for a lower threshold for blood transfusion in elderly patients who underwent surgery for femoral neck fractures. D’Amore et al. used a postoperative hemoglobin (Hb) level of <9 g/dL on the first postoperative day to prevent later complications in this specific population [30]. Patil et al. chose to transfuse patients with Hb levels of 80–100 g/L if they had a history of cardiovascular disease [7].

Despite adopting a higher Hb level for transfusions, we concluded that routine blood tests following total hip arthroplasty for femoral neck fractures are unnecessary in the absence of intraoperative complications. This higher Hb level threshold further supports our conclusion.

Among the 170 study patients, 96.59% maintained postoperative Hb levels above 8.5 g/dL (Table 1). However, 17% of these patients received blood transfusions after surgery, indicating that a significant portion of transfusions may have been unnecessary. The data suggests that many transfusions were administered by less experienced residents, possibly due to a lack of refined clinical judgment. This observation mirrors findings previously reported by Salem-Schatz et al. [31].

Based on the findings of our study, we have revised our department’s guidelines for blood transfusions in elderly patients undergoing THA. Our revised protocol now indicates transfusions for individuals with hemoglobin levels below 8.5 g/dL or those with higher hemoglobin levels who exhibit symptoms of anemia.

Lastly, and crucial to our study, we found that only 3.41% of patients had Hb levels below 8.5 g/dL post-surgery, indicating the need for closer monitoring in these cases. These findings suggest that fast-tracking could be doable even when treating femoral neck fractures within specific demographics. Our results suggest that 96% of our patients could have been safely discharged on the day of surgery, without prolonged hospitalizations or extensive Hb monitoring. It is critical to emphasize that the majority of fast-track approaches following THA are typically applied to elective patients with hip osteoarthritis. However, our study suggests that similar outcomes can be achieved in cases of THA for displaced femoral neck fractures by adhering to the NICE guidelines [15].

Our findings have significant economic and healthcare implications, particularly regarding the potential cost savings from avoiding post-operative blood tests and possibly reducing hospital stays. We suggest considering the adoption of fast-tracking protocols for healthy elderly patients when their surgeries are performed flawlessly. In these cases, a specialized outpatient team could conduct the routine post-operative blood tests at the patient’s home.

The current study has some limitations. The study is retrospective. We had a comparably small sample size, which limited the power and increased the risk of a type II error. Consequently, the conclusions and recommendations should be approached with caution. It is important to recognize that we did not adhere to the standard recommendations for blood transfusion following THA. Instead, we used lower thresholds due to the unique nature of the study’s cohort of frail elderly patients. The surgeries were conducted exclusively by three attending surgeons within a single public hospital, potentially limiting the generalizability of our findings. There was no strict protocol that determined the criteria for blood transfusions. A future prospective multi-center study with a strict protocol that evaluates the value of postoperative blood tests is needed to address these limitations effectively. 

## 5. Conclusions

Based on our findings, postoperative blood count testing may be unnecessary for patients who have undergone total hip arthroplasty (THA) for displaced femoral neck fractures, especially in the absence of complications or significant underlying health conditions.

## Figures and Tables

**Table 1:** Characteristics and univariable analysis of patients divided by postoperative hemoglobin levels.

	PoOHb > 8.5 (n = 170, 96.59%)	PoOHb ≤ 8.5 (n = 6, 3.41%)	Total (n = 176, 100%)	*p* Value
Age	69.23 ± 7.98	65 ± 11.85	69.09 ± 8.13	0.424
Sex male	65 (38.2%)	2 (33.3%)	67 (38.1%)	1.000
Time duration from admission to surgery				0.328
On the first day of admission	95 (55.9%)			
On the second day of admission	43 (25.3%)			
On the third day of admission	22 (12.9%)			
On the fourth day of admission or later	10 (5.9%)			
Surgery duration (minutes)	111.76 ± 38.41	108 ± 24.17	111.63 ± 37.98	0.728
PrOHb	13.1 ± 1.36	12.5 ± 1.71	13.09 ± 1.37	0.428
PoOHb	10.62 ± 1.06	7.85 ± 0.43	10.53 ± 1.16	<0.001
Hb difference following surgery	2.48 ± 1.18	4.65 ± 1.78	2.56 ± 1.26	0.031
Anesthesia general (vs. spinal)	98 (57.6%)	5 (83.3%)	103 (58.5%)	0.403
Hypertension	86 (50.6%)	1 (16.7%)	87 (49.4%)	0.211
Dyslipidemia	73 (42.9%)	2 (33.3%)	75 (42.6%)	1.000
Diabetes mellitus	39 (22.9%)	0 (0.0%)	39 (22.2%)	0.341
Respiratory disease	16 (9.4%)	0 (0.0%)	16 (9.1%)	1.000
Clotting disturbance	4 (2.4%)	0 (0.0%)	4 (2.3%)	1.000
Peripheral vascular disease	17 (10.0%)	0 (0.0%)	17 (9.7%)	1.000
Ischemic heart disease	28 (16.5%)	0 (0.0%)	28 (15.9%)	0.591
Smoking	26 (15.3%)	2 (33.3%)	28 (15.9%)	0.244
BMI	29 ± 3.58	22.92 ± 2.73	28.55 ± 2.78	0.077
Blood transfusion after surgery	28 (16.5%)	5 (83.3%)	28 (15.9%)	<0.001

PrOHb—preoperative hemoglobin; PoOHb—postoperative hemoglobin; Hb—hemoglobin; BMI—basal metabolic index.

**Table 2:** Characteristics and univariable analysis of patients divided by administration of blood transfusion.

Variable	Did Not Receive Blood Transfusion (n = 143, 81.25%)	Received Blood Transfusion (n = 33, 18.75%)	Total (n = 176, 100%)	*p* Value
Age	68.80 ± 8.41	70.33 ± 6.75	69.09 ± 8.13	0.267
Sex male	87 (60.8%)	22 (66.7%)	109 (61.9%)	0.534
Time duration from admission to surgery				0.471
On the first day of admission	78 (54.5%)	21 (63.6%)	99 (56.3%)	
On the second day of admission	38 (26.6%)	5 (15.2%)	43 (24.4%)	
On the third day of admission	19 (13.3%)	4 (12.1%)	23 (13.1%)	
On the fourth day of admission or later	8 (5.6%)	3 (9.1%)	11 (6.3%)	
Surgery duration (minutes)	109.18 ± 37.18	122.24 ± 40.14	111.63 ± 37.98	0.095
PrOHb	13.19 ± 1.36	12.63 ± 1.33	13.09 ± 1.37	0.035
PoOHb	10.79 ± 1.02	9.38 ± 1.06	10.53 ± 1.16	<0.001
Hb difference following surgery	2.4 ± 1.17	3.25 ± 1.45	2.56 ± 1.26	0.003
Anesthesia general (vs. spinal)	22 (15.4%)	11 (33.3%)	33 (18.7%)	0.292
Hypertension	71 (49.6%)	16 (48.5%)	87 (49.4%)	0.904
Dyslipidemia	61 (42.7%)	14 (42.4%)	75 (42.6%)	0.981
Diabetes mellitus	33 (23.1%)	6 (18.2%)	39 (22.2%)	0.542
Respiratory disease	16 (11.2%)	0 (0.0%)	16 (9.1%)	0.045
Clotting disturbance	3 (2.1%)	1 (3.03%)	4 (2.3%)	0.568
Peripheral vascular disease	15 (10.5%)	2 (6.1%)	17 (9.7%)	0.743
Ischemic heart disease	22 (15.38%)	6 (18.18%)	28 (15.91%)	0.692
Smoking	23 (16.08%)	5 (15.15%)	28 (15.91%)	0.895
BMI	25.84 ± 4.11	25.75 ± 4.29	25.62 ± 4.13	0.345

PrOHb—preoperative hemoglobin; PoOHb—postoperative hemoglobin; Hb—hemoglobin; BMI—basal metabolic index.

## Data Availability

The raw data supporting the conclusions of this article will be made available by the authors on request.
